# Calcium and Vitamin D Supplementation with and Without Collagen on Bone Density and Skin Elasticity in Menopausal Women—A Randomized Controlled Study

**DOI:** 10.3390/clinpract15090168

**Published:** 2025-09-15

**Authors:** Acharaporn Duangjai, Jukkarin Srivilai, Sawitree Nangola, Doungporn Amornlerdpison

**Affiliations:** 1 Unit of Excellence in Research and Product Development of Coffee, Division of Physiology, School of Medical Sciences, University of Phayao, Phayao 56000, Thailand; 2 Research and Innovation Center in Cosmetic Sciences and Natural Products, Department of Cosmetic Sciences, School of Pharmaceutical Sciences, University of Phayao, Phayao 56000, Thailand; jukkarin.sr@up.ac.th; 3 Department of Medical Technology, School of Allied Health Sciences, University of Phayao, Phayao 56000, Thailand; sawitree.na@up.ac.th; 4 Center of Excellence in Agricultural Innovation for Graduate Entrepreneur, Maejo University, Chiang Mai 50290, Thailand; doungporn_a@mju.ac.th; 5 Faculty of Fisheries Technology and Aquatic Resources, Maejo University, Chiang Mai 50290, Thailand

**Keywords:** calcium, collagen, bone density, skin elasticity, menopausal women

## Abstract

Background/Objectives: Menopause leads to estrogen deficiency, which negatively affects bone density, skin integrity, and hair health in women. This study aimed to evaluate the effects of fish-derived collagen peptides, calcium, and vitamin D3 supplementation on body composition, bone turnover markers, skin condition, and hair loss in menopausal women. Methods: Participants were randomized into four groups: placebo (G01), 1000 mg calcium + 400 IU vitamin D3 (G02), 5 g collagen (G03), and 1000 mg calcium + 400 IU vitamin D3 + 5 g collagen (G04). Participants received daily supplementation for six months. Body composition, biochemical bone markers (P1NP, BAP, osteocalcin), skin hydration, elasticity, transepidermal water loss (TEWL), and hair loss were assessed at baseline and follow-ups. Results: No significant changes were observed in body composition or bone biomarkers including P1NP, BAP, and osteocalcin across groups. Serum creatinine, ALT, and AST levels remained within normal ranges. Serum calcium levels remained stable, and urinary calcium excretion slightly increased in calcium-supplemented groups, indicating no adverse effects on kidney or liver function. G02 and G04 exhibited slightly decreased serum calcium levels compared to G01 and G03. However, G04 showed significantly improved skin hydration by 23% and skin elasticity by 8.52% compared to baseline after six months, whereas the placebo group showed negligible changes. G03 also showed notable improvement in elasticity by 12.23%, indicating collagen’s dominant role. The G02, G03, and G04 also significantly retarded hair shedding compared to the placebo (G01) group. TEWL did not significantly change in any group. Conclusions: These findings suggest that six-month supplementation with collagen peptides, particularly when combined with calcium and vitamin D, improves skin hydration and elasticity in menopausal women.

## 1. Introduction

Menopause plays a major role in altering a woman’s body composition. It typically leads to increased fat accumulation, especially visceral fat around the organs, and a reduction in lean muscle mass [1,2]. Furthermore, estrogen deficiency triggers a rapid decline in skin collagen, with women potentially losing up to 30% of dermal collagen within the first five years after menopause. The marked reduction in collagen profoundly affects skin architecture, resulting in enhanced extensibility, diminished elasticity, and heightened fragility, thereby contributing to the progression of cutaneous aging [3]. The decline in estrogen levels contributes to bone density loss, raising the risk of osteoporosis [4]. Moreover, menopause-related skin and hair changes can really affect a woman’s quality of life [4]. Therefore, calcium and collagen supplementations are a valuable option for improvement in postmenopausal women.

A calcium–collagen chelate supplement enhanced bone mineral density (BMD) and helped accelerate bone formation to outpace bone resorption in postmenopausal women with osteopenia [5]. Calcium and vitamin D are vital for bone health, particularly for women during and after menopause, as they reduce bone loss. A daily intake of 1200 mg of calcium is recommended for most postmenopausal women [6]. However, the effectiveness of calcium supplementation depends not only on the dosage but also on its bioavailability, which varies across calcium compounds [7]. The body’s ability to absorb calcium varies significantly depending on the chemical form of the calcium compound, particularly its water solubility. Organic calcium salts, like calcium lactate citrate and calcium lactate malate, dissolve much better than common calcium carbonate [8]. However, they tend to be more expensive than less soluble options such as calcium carbonate. Interestingly, the natural hydroxyapatite (HA) extracted from fish bones closely resembles the human bone in its calcium and phosphorus-rich composition and structure, and it is a promising material for biomedical applications [9].

This clinical trial was to assess the effects of calcium from bone fish either alone or in combination with a collagen peptide product from fish skin on body composition, bone biomarkers, and dermatological assessments in menopause. In the present randomized study, we aimed to examine and compare the efficacy, as represented by the changes in body composition, bone biomarkers procollagen type I N-terminal propeptide (P1NP), alkaline phosphatase bone isoenzyme, osteocalcin, skin hydration, elasticity, and hair condition of 6-month supplementation of calcium and vitamin D supplementation with and without collagen in menopausal women.

## 2. Materials and Methods

The study protocol was approved by the Institutional Review Board of University of Phayao and written informed consent were obtained before participating in this study (No. HREC-UP-HSST 1.3/035/66). The study was registered at Thaiclinicaltrials.org (TCTR20231021002). Subjects could withdraw from the study at any time.

### 2.1. Materials

All supplements used in this study were standardized for quality. Hydrolyzed collagen (5 g per dose) and biocalcium (providing 1000 mg of elemental calcium per dose) were obtained from the Center of Excellence of Agricultural Innovation for Graduate Entrepreneurs, Maejo University, Thailand. These were extracted from the skin and bone of the Buk-Siam Maejo hybrid catfish (*Pangasianodon gigas* × *Pangasianodon hypophthalmus*), respectively. Vitamin D3 (cholecalciferol, 400 IU per dose) was sourced from DSM Nutritional Products Ltd., Kaiseraugst, Switzerland, and complied with established dietary supplement quality standards. All supplements used in this study were standardized for quality. The calcium and vitamin D3 were provided either separately or in combination with collagen, depending on group assignment. All products were in powder or tablet form and were administered orally once daily for 6 months. The supplements were packaged and blinded to ensure double-blind conditions throughout the trial.

### 2.2. Participants

A total of 80 menopausal women were screened following the inclusion criteria: Female menopausal volunteers aged between 45 and 60 years were eligible to participate in the study. Participants must be in good health, with no swallowing difficulties, and free from severe diseases or complications. They should not have a history of receiving estrogen-suppressing medications. Volunteers must not have a history of regular alcohol consumption, which is defined as consuming alcoholic beverages such as liquor or beer more than once per week or using illicit drugs. Additionally, they must not have a history of smoking or must have quit smoking for at least 30 days prior to the study. The following exclusion criteria were used: participants must not have used anti-inflammatory drugs, antibiotics, or steroid medications within 14 days before receiving the research product and must commit to refraining from their use throughout the study. Similarly, they must not have consumed dietary supplements, vitamins, minerals, herbal products, or hormone replacement therapy (HRT) within 14 days prior to the study and must not use them during the study period. Individuals who have received filler injections, stem cell injections, fat cell injections, or botulinum toxin injections in the test area are not eligible to participate. Additionally, volunteers must not have participated in any other clinical studies within the past 30 days. Blood donation within 90 days before enrollment in the study is not permitted. Finally, volunteers must be willing to participate in the study and sign an informed consent form, acknowledging their understanding of the study’s procedures and requirements.

### 2.3. Study Design

**Figure 1:**
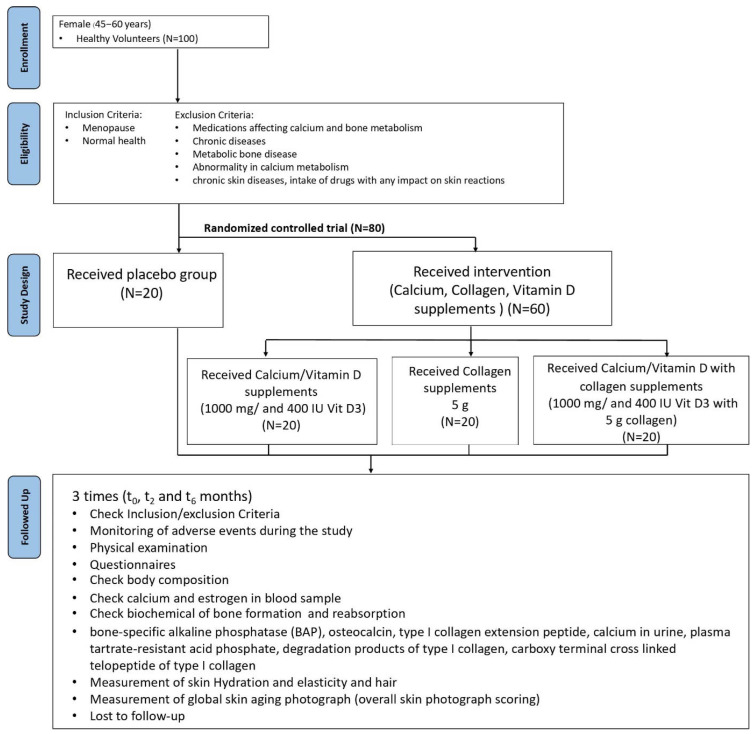
Flowchart diagram of study design, participant allocation, and follow-up in a randomized controlled trial on supplementation effects in menopausal women.

The study was carried out in a randomized, placebo-controlled trial as shown in Figure 1. Eighty healthy menopausal women recruited, seventy-nine women completed the study. The sample size was estimated using the conventional formula for continuous outcomes with equal group sizes, based on data from [10]. The calculation indicated a minimum of 17 participants per group, and to account for potential attrition, we increased the sample size by 20% following methodological recommendations [11]. Participants were randomized into four groups. Group 01 (G01; control) consisted of 20 subjects who received placebo. Group 02 (G02; intervention) consisted of 20 subjects who received 1000 mg calcium and 400 IU vitamin D3. Group 03 (G03; intervention) consisted of 20 subjects who received 5 g collagen. Group 04 (G04; intervention) consisted of 20 subjects who received 1000 mg calcium, 400 IU vitamin D3, and 5 g collagen. The dosage of the supplements was determined based on previous clinical studies which demonstrated both efficacy and safety within this dose range [5,6,12]. The selected dose was considered appropriate to elicit measurable effects on the target outcomes without exceeding the recommended upper intake levels.

### 2.4. Measurements

Participants were followed up at three time points: baseline (T_0_), 2 months (T_2_), and 6 months (T_6_). The 2-month follow-up was used to evaluate early response and adherence, while the 6-month follow-up assessed the sustained effects on the primary outcomes. At each visit, the inclusion and exclusion criteria were re-evaluated, and adverse events were monitored to ensure participant safety. A physical examination was conducted, and participants were asked to complete questionnaires to assess subjective outcomes. Body composition, including bone mass and related parameters, was measured. Blood samples were collected to determine calcium, creatinine, alanine transaminase (ALT), aspartate transaminase (AST), estradiol hormone, biochemical markers of bone formation, and resorption including bone-specific alkaline phosphatase (BAP), osteocalcin, and procollagen Type I N-terminal propeptide (P1NP). Urine samples were collected to determine the level of calcium. Dermatological assessments were also performed, including measurement of skin hydration, elasticity, and hair condition. In addition, standardized global skin aging was evaluated using photographic scoring. Data regarding participant retention and any loss to follow-up were also recorded throughout the study period.

### 2.5. Anthropometric Measurements

Anthropometric and body composition measurements were conducted using a Bioelectrical impedance analysis (BIA) device [BC720, ACCUNIQ; SELVAS Healthcare, Inc., Daejeon, Republic of Korea], which provides a non-invasive, reliable estimation of body composition. Prior to the measurement, participants were instructed to fast for at least 2 h, void their bladder, and refrain from intense physical activity for 12 h to minimize fluctuations in body water and ensure accuracy. Each participant stood barefoot on the device platform and grasped the handles with both hands according to the manufacturer’s standard protocol. Participants were asked to remain still in an upright posture while lightly holding the hand electrodes with their arms slightly abducted from the trunk. The measurement took approximately 5 min to complete. The BIA device analyzed multiple components of body composition, including lean body mass (L.B.M.), soft lean mass (SLM), total body water (TBW), protein mass, mineral content, mass body fat (M.B.F.), skeletal muscle mass (S.M.M.), body mass index (BMI), percentage body fat (P.B.F.), waist-to-hip ratio (W.H.R.), and visceral fat area (V.F.A.).

### 2.6. Sample Collection and Biochemical Marker Measurements

Blood and urine samples were obtained from each subject at baseline (T0), 2-month (T2), and 6-month (T6) time points for the analysis of biochemical markers. Midstream urine was collected in sterile containers. After overnight fasting, a venous blood sample (10 mL) was obtained by a well-trained medical technologist using standard procedure and divided into two tubes containing EDTA and clot activator. Blood samples were centrifuged at 3500× *g* for 15 min at 4C. Plasma, serum, and urine samples were collected, aliquoted, and sent to the laboratory under cold chain delivery condition.

Levels of urine calcium, serum calcium, creatinine, alanine transaminase (ALT), and aspartate transaminase (AST) levels were measured using the colorimetric method (Abbott Laboratories, Wiesbaden, Germany). Bone-specific alkaline phosphatase (BAP) concentration was measured using the Para-nitrophenyl phosphate (p-NPP) method (Abbott Laboratories, Wiesbaden, Germany). Bone turnover marker P1NP and osteocalcin hormones were measured using an electrochemiluminescent immunoassay (Roche diagnostics, Penzberg, Germany). The estradiol hormone was measured using a chemiluminescent microparticle immunoassay (Abbott Laboratories, Wiesbaden, Germany).

### 2.7. Skin Measurement Instrumentation

For transepidermal water loss (TEWL), a TEWL probe of DermalLab^®^ Combo a (Cortex Technology, Aalborg, Denmark), measured the rising chamber humidity as water evaporated from the skin. The facial area was randomly measured on Zygomatic area. Elasticity was measured using an elasticity probe of DermalLab^®^ Combo a (Cortex Technology, Aalborg, Denmark), based on the principle of vertical suction applied on the surface of scar. The probe has a measuring aperture of 10 mm diameter and adheres to the skin by a double adhesive sticker. The facial area was randomly measured on the orbicularis area. For hair strength was measured by the combing test, analyzing the hair count [13]; subjects did not shampoo on the day of measuring and combed the hair for 60 s over a sheet of contrasting color to the hair, starting with the comb at the back top of the scalp and moving the comb forward to the front of the scalp 20 times. A global photograph was used to evaluate overall visual differences in facial skin, with the camera (Canon EOS 70d) position and lighting conditions standardized and maintained at identical coordinates for each assessment to ensure consistency and reproducibility.

### 2.8. Statistical Analysis

Data are expressed as mean ± standard deviation. The differences within the group of the not normally distributed data were evaluated via the Komogorov–Smirnov test while the normally distributed data were evaluated using the unpaired *t*-test. The Kruskal–Wallis test was used to evaluate differences between groups. The statistical tests of biochemical data were performed using Graphad Prism version 6.01. The value of *p* < 0.05 indicated the statistical significance.

## 3. Results

### 3.1. Body Composition

The preliminary physical assessment and segmental body composition analysis are presented in Table 1, Table 2, Table 3 and Table 4. The findings showed that participants across all four groups had similar baseline characteristics. Height ranged between 153 and 155 cm, body weight (comprising fat, muscle, bone, and water) ranged from 52 to 57 kg, and age ranged from 52 to 56 years. The analysis of body composition parameters, including standard weight, lean body mass (L.B.M.), soft lean mass (SLM), total body water (TBW), protein mass, mineral content, mass body fat (M.B.F.), skeletal muscle mass (S.M.M.), body mass index (BMI), percentage body fat (P.B.F.), waist-to-hip ratio (W.H.R.), and visceral fat area (V.F.A.), revealed comparable values among the groups. Furthermore, segmental analysis of lean mass (LEFT ARM S.L.M., RIGHT ARM S.L.M., LEFT LEG S.L.M., RIGHT LEG S.L.M., TRUNK S.L.M.) and fat mass (LEFT ARM M.B.F., RIGHT ARM M.B.F., LEFT LEG M.B.F., RIGHT LEG M.B.F., TRUNK M.B.F.) showed no significant differences between the groups. Other measured variables such as basal metabolic rate (B.M.R.), calorie expenditure (CALORY), and body cell mass (B.C.M.) were also consistent across all groups. After six months of intervention with the investigational products, no statistically significant changes were observed in the aforementioned body composition parameters across the groups.

### 3.2. Biochemical Parameters for Evaluation of the Effect of Product Among the Four Groups

The evaluation of the side effects of consuming the product on kidney and liver function was assessed through serum creatinine, ALT, and AST. The level of these three parameters of all the participants was shown within the normal range at all three time points (Table 5). There were no volunteers who had values exceeding the normal range and indicated that the consumption of the product in this study did not affect liver or kidney function.

The level of calcium as shown in Table 6 revealed that the average urinary calcium levels (urine calcium) in the placebo group (G01) decreased more than in the other groups. The groups receiving collagen (G03) and calcium/vitamin D supplementary with collagen (G04) had levels close to their baseline values. It was also found that urinary calcium excretion increased in the group that received calcium/vitamin D as a combination. Considering this result alongside the total serum calcium levels, it was observed that the groups receiving products containing calcium, both calcium/vitamin D (G02), and calcium/vitamin D supplementary with collagen (G04), showed a slightly decrease in calcium levels with no statistical difference. In contrast, the placebo (G01) and collagen (G03) groups had a statistically significant decrease in average serum calcium levels (*p* < 0.004). This suggests that the intake of products containing calcium/vitamin D may help improve calcium absorption.

The evaluation of specific biomarkers for bone formation was analyzed in all the groups as shown in Table 7. At baseline, all markers including P1NP, osteocalcin, and BAP were similar between study groups. After 6 months of supplementation, P1NP and osteocalcin slightly decreased in the groups received calcium/vitamin D (G02), collagen (G03), and calcium/vitamin D supplementary with collagen (G04) with no statistical difference in both between groups and within groups. Therefore, BAP concentration slightly increased in all groups with no statistical changes.

### 3.3. Skin Barrier Function

**Figure 2:**
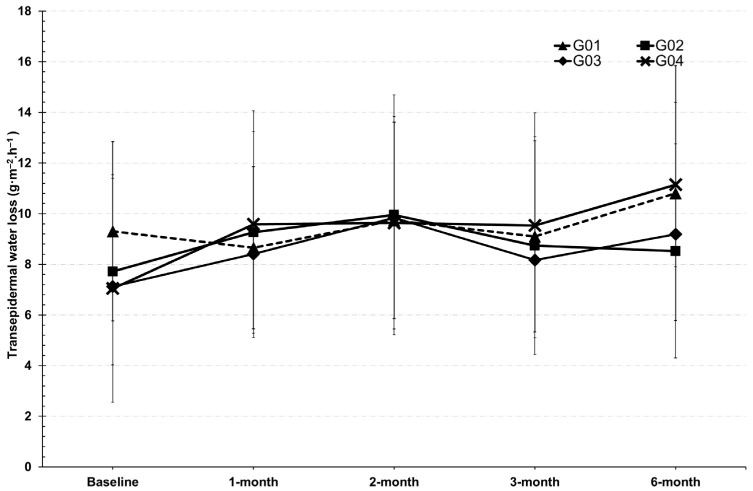
Transepidermal water loss (TEWL) of tested groups.

The rate of water evaporates from the skin, known as transepidermal water loss (TEWL), indicates the function of skin barrier to retain moisture, particularly in the stratum corneum, which is composed of lipids and keratin proteins. A high TEWL indicates that the skin barrier has been weakened, thinned, damaged, or impaired, as observed with skin irritation or injury. In this study, TEWL showed unchanged from baseline after six months of continuous product consumption, with no significant differences observed within or between the test groups, as shown in Figure 2.

### 3.4. Skin Hydration

**Figure 3:**
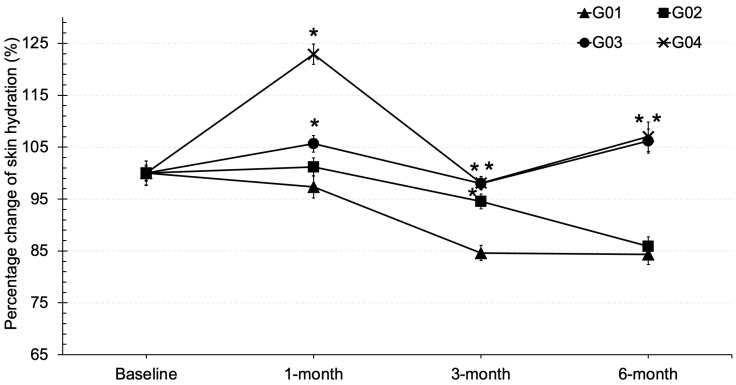
Skin hydration in percentage change from baseline in various time courses of tested groups; * stands for significantly different from placebo (*p* < 0.05).

The skin hydration value refers to the amount of water present in the viable skin layer of the epidermis, which lies beneath the stratum corneum. Higher value indicates good water retention and healthy skin. In this experiment, skin hydration was measured on the cheek area (zygomatic major or C-zone) of volunteers and expressed as a percentage change from baseline. The results showed that the average percentage change in skin hydration after one month of use increased by approximately 23% in the group that received collagen + vitamin D + calcium (G04) and by 5% in the group that received only collagen (G03), compared to pre-treatment levels. These increases were significantly different from baseline (*p* < 0.05) after one month of testing. In contrast, the group that received calcium + vitamin D showed no improvement in skin hydration compared to baseline (Figure 3) and followed the same trend as the placebo group. The study demonstrated the effectiveness of the test products, particularly in groups G03 and G04, after six months of continuous use, as they exhibited significantly better increases in skin hydration compared to groups G01 and G02 (*p* < 0.05).

### 3.5. Skin Elasticity

**Figure 4:**
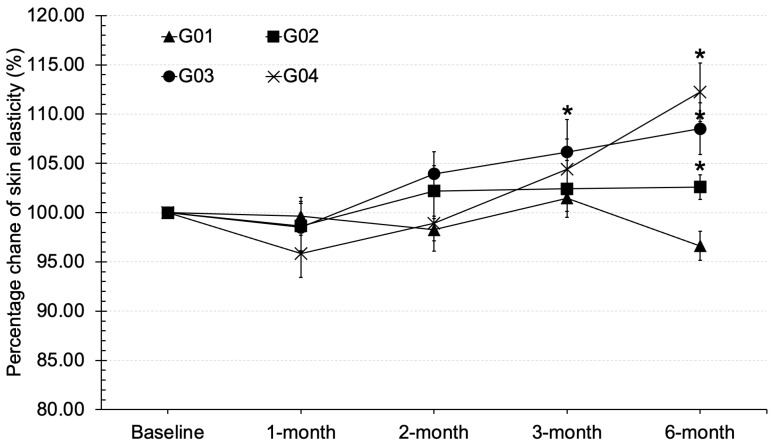
Skin elasticity in percentage change from baseline in various time courses of tested groups; * stands for significantly different from placebo (*p* < 0.05).

In this study, the elasticity of the skin was examined when treated with calcium + vitamin D (G02), collagen (G03), collagen + vitamin D + calcium (G04), and the placebo group (G01). Skin elasticity is a desirable property, with higher values indicating better dermal integrity, as fibroblast cells in the dermis can produce essential protein fibers like elastin and collagen. The experiment analyzed skin elasticity values as a percentage change from baseline as shown in Figure 4. After one month of testing, there were no significant differences between the groups. However, after two months, an upward trend in skin elasticity was observed, becoming more apparent after three months of product use. By month six, the G04 and G03 groups showed significantly higher skin elasticity, at 108.52 ± 0.03% and 112.23 ± 0.05%, respectively, compared to the control group. However, the effects were not as pronounced as those seen with collagen, as observed in the G02 group, which maintained better skin elasticity than the placebo group. The findings from this study highlight the critical role of long-term supplementation in enhancing skin elasticity. The delayed yet significant improvements seen after three to six months of supplementation, particularly in the groups receiving collagen (G03 and G04), underscore the importance of sustained product use for optimal results.

### 3.6. Hair Falling

**Figure 5:**
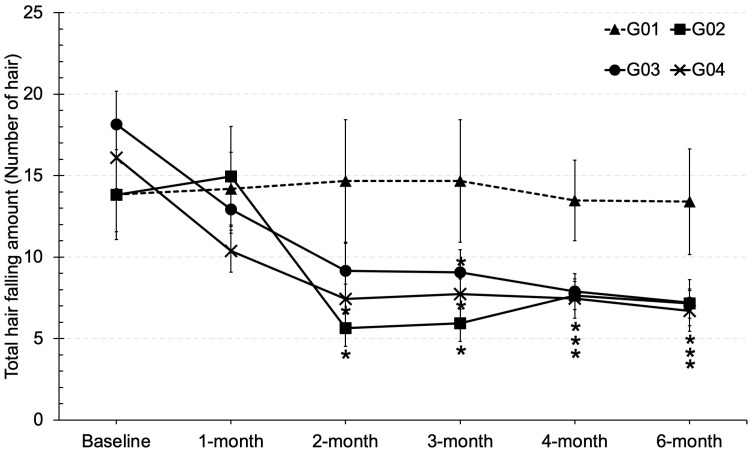
Total hair count in various time courses of tested groups; * stands for significantly different from placebo (*p* < 0.05).

In this study, the effects of calcium + vitamin D (G02), collagen (G03), collagen + vitamin D + calcium (G04), and a placebo (G01) on hair falling were investigated among menopausal women. Hair loss is a common problem during menopause, primarily resulting from the decline in estrogen levels. This hormonal reduction contributes to de-creased keratin synthesis, follicular miniaturization, and ultimately, increased hair shedding. At baseline, the mean number of shed hairs did not differ significantly among the four groups, with values ranging from 13.8 ± 2.2 to 18.1 ± 2.0 as shown in Figure 5. After three months of supplementation, participants in the G02, G03, and G04 groups demonstrated a statistically significant reduction in hair loss compared with the placebo group. The mean hair shedding values at this time point were 5.9 ± 1.1, 9.0 ± 1.4, and 7.7 ± 0.9 for G02, G03, and G04, respectively. Importantly, these beneficial effects were sustained throughout the six-month study period. These findings provide clinical evidence supporting the role of calcium, collagen, and vitamin D in attenuating menopausal hair loss.

## 4. Discussion

The present study investigated the effects of calcium and vitamin D supplementation, with or without fish-derived collagen peptides, on body composition, bone biomarkers, and dermatological parameters in menopausal women. Adequate intake of calcium and vitamin D, in combination with collagen supplementation, may play a significant role in enhancing overall physical health. This is particularly relevant to internal body health, as determined through body weight analysis, fat mass, and muscle mass appropriate to age and sex. A body age exceeding chronological age suggests the need for reduced fat, increased lean protein mass, and improved metabolic rate to support better physiological function. Our findings indicate that calcium and vitamin D, either alone or combined with collagen, did not significantly influence body composition indices such as body weight, lean body mass (LBM), muscle mass (soft lean mass—SLM), total body water (TBW), protein mass, bone mineral content (Mineral), mass body fat (MBF), skeletal muscle mass (SMM), body mass index (BMI), percentage body fat (PBF), waist-to-hip ratio (WHR), visceral fat area (VFA), basal metabolic rate (BMR), total calorie expenditure, or body cell mass (BCM) during the 6-month intervention. However, postmenopausal women with low bone mineral density (osteopenia) who were randomly assigned to receive calcium, vitamin D, and collagen peptides (intervention group), or only calcium and vitamin D (control group), experienced improved bone health with collagen peptide supplementation. The benefits of calcium, vitamin D, and collagen on bone turnover markers, some studies indicate that the effects on bone formation markers are less consistent. For instance, vitamin D supplementation alone showed variable results in influencing bone formation markers such as BAP [14]. This suggests that while these supplements are beneficial, their effects may vary based on individual factors and specific supplementation regimens. Most studies did not detect significant changes in circulating levels of plasma bone metabolism marker after supplementation, especially bone formation markers including P1NP, osteocalcin, and BAP [15] and serum calcium level [16]. Calcium supplements, particularly when combined with vitamin D, do not significantly alter serum calcium levels in postmenopausal women, as serum calcium levels did not vary significantly despite prolonged supplementation, while urinary calcium levels increased significantly [16]. In terms of bone health, supplementation with calcium, vitamin D, and collagen peptides showed a favorable trend in bone turnover markers, particularly a reduction in P1NP levels compared to calcium and vitamin D alone. This suggests that collagen peptides may enhance the positive effects of calcium and vitamin D on bone turnover [12]. Another study demonstrated that fortified milk containing 1200 mg of calcium and vitamin D significantly reduced urinary excretion of bone resorption markers, indicating a positive effect on bone turnover [17]. A systematic review of randomized controlled trials on the role of food fortification with vitamin D and calcium revealed that daily consumption of soft plain cheese fortified with 2.5 µg of vitamin D3 and 302 mg of calcium for 4 weeks resulted in a mean increase of 15.9 ng/mL in P1NP levels compared to baseline. However, yogurt fortified with 10 µg of vitamin D3 and 800 mg of calcium did not change P1NP values after 8 weeks. Overall, the interventions improved bone resorption but did not significantly enhance bone formation in postmenopausal women [18]. The study of combining calcium, vitamin D, and collagen may enhance bone health beyond individual supplements and reduce bone resorption and increase formation markers, suggesting a balanced bone turnover [12,19].

Collagen, particularly in its hydrolyzed form, provides superior digestibility and bioavailability compared to native collagen as it is broken down into smaller peptides and free amino acids such as glycine and proline. These amino acids serve as essential precursors for the synthesis and remodeling of connective tissue proteins, thereby supporting the structural integrity of skin, hair, and muscle [20]. However, collagen supplementation alone may have limited influence if not complemented by cofactors that regulate epidermal differentiation and hydration. Vitamin D plays a critical role in this regard. It enhances intracellular calcium signaling and activates calcium receptors, processes that drive keratinocyte differentiation and accelerate epidermal turnover [21]. At the same time, vitamin D stimulates the synthesis of key natural moisturizing factors, including glycosylceramides, involucrin, loricrin, and filaggrin [22]. Collectively, these actions improve barrier function and skin hydration. Thus, the synergistic contribution of hydrolyzed collagen and vitamin D lies in collagen remodeling at the dermal level combined with improved keratinocyte function and epidermal moisturization. Dermatological outcomes revealed a more consistent effect. Collagen supplementation significantly improved skin hydration and elasticity compared with placebo, confirming its role in enhancing dermal structure. TEWL value reflects the ability of skin barrier function to retain moisture, especially in the lipid- and keratin-rich stratum corneum. According to the results, oral administration of collagen, calcium, and vitamin D did not appear to influence keratin synthesis in the epidermal layer, as evidenced by TEWL values that remained comparable to baseline. This suggests that the primary site of action for these compounds is more likely within the dermal layer, where collagen and elastin are predominant. Consequently, collagen supplementation may have exerted only a minimal impact on the epidermal parameters assessed in this study. Furthermore, the study indicates that collagen supplementation over six months had no effect on reducing TEWL in menopausal participants. This suggests that a higher intake of collagen may be required in future studies to enhance stratum corneum function in menopausal participants. However, TEWL rates across all test groups range from 2 to 14 g/m^2^/h at baseline, which falls within the normal range for skin [23]. As for the skin hydration parameter, the groups that received collagen significantly improved skin hydration, the study highlighted collagen as a key factor in improving skin hydration. This finding is consistent with previous research [21]. Moreover, the group receiving vitamin D, calcium, and collagen showed enhanced skin hydration, likely due to vitamin D play role in increasing the synthesis of moisturizing substances in the skin, such as glycosylceramide, involucrin, loricrin, and filaggrin, which are natural moisturizing factors. Vitamin D also enhances intracellular calcium levels, induces calcium receptors, and stimulates keratinocyte differentiation, promoting skin cell turnover and increasing moisture [21,22]. However, a general decline in hydration was observed across all groups at the 3-month visit, possibly due to external factors like weather affecting skin condition. Furthermore, improvements in skin hydration could have been influenced by individual differences in daily water intake, an important parameter that was not controlled in this study and may have directly contributed to variability in the hydration outcomes. Interestingly, skin elasticity significantly improved after two months of continuous use of collagen and vitamin D (G03 and G04), suggesting that prolonged supplementation particularly beyond three months is necessary for noticeable effects. This aligns with previous research [23], which indicates that the synthesis of structural protein fibers in the dermis requires over three months. The combination of collagen with vitamin D and calcium may further support skin elasticity, likely through vitamin D’s role in promoting keratinocyte differentiation and epidermal strengthening [22]. However, these effects were less pronounced than those of collagen alone. For instance, the G02 group (collagen only) showed greater improvements than the placebo, reinforcing collagen as the primary contributor to enhanced elasticity. These findings emphasize the importance of long-term supplementation, particularly with collagen, to achieve visible improvements in skin structure. Collagen, a key component of the extracellular matrix, plays a central role in restoring elasticity over time. Vitamin D and calcium appear to provide synergistic benefits by supporting skin cell turnover and barrier integrity, though to a lesser extent. Overall, this study supports the extended use of collagen alone or in combination with vitamin D and calcium as an effective strategy for improving skin elasticity, especially in aging populations. This study also evaluated the effect on hair fall reduction in menopausal volunteers and found a statistically significant improvement after three months compared to the placebo. Our results are consistent with existing research that highlights the role of vitamin D in hair health. Injectable vitamin D has been shown to promote hair growth and prevent hair loss in individuals undergoing chemotherapy [24]. The mechanism of vitamin D involves reducing hair falling and stimulating new hair growth by influencing hair follicle cells and inducing the production of proteins such as Wnt10b, ALPL, and TGF-B2 [25]. This suggests that vitamin D might play a crucial role in maintaining hair follicle health and supporting the hair growth cycle. Furthermore, some findings support the potential benefits of collagen in the reduction in hair loss among postmenopausal women. Previous research indicates that collagen may enhance the synthesis of protein fibers in hair follicle cells, which can improve follicle health and reduce hair loss [26]. The role of collagen supporting the structural integrity of hair follicles could be particularly beneficial during menopause when hair thinning and loss are common. Overall, the study provides evidence that both vitamin D and collagen supplements can positively impact hair health in menopausal individuals. Our results suggest that these interventions may help manage hair falling during menopause, aligning with earlier research on their respective benefits. This study demonstrates several key strengths. It assessed the effects of collagen supplementation both alone and in combination with vitamin D and calcium, allowing a comprehensive evaluation of potential synergistic benefits. The study specifically targeted early menopausal women, a population particularly susceptible to hair thinning and decreased skin elasticity, enhancing the clinical relevance of the findings. Multiple outcomes including skin elasticity, hair fall, and biochemical markers of bone health were measured, providing a holistic view of the interventions’ effects. Future studies should explore the long-term effects and optimal dosages of these supplements to maximize their benefits for hair health. The findings suggest that calcium and collagen peptides may have more significant effects when combined with adequate macronutrient intake. However, the study population was limited to menopausal women, and the short-term duration may have limited observable changes in bone density and skin condition. Further long-term studies are necessary to evaluate sustained effects, particularly in combination with dietary control or regular exercise. Potential confounding factors such as variations in lifestyle and dietary intake, though minimized, cannot be entirely excluded. Additionally, the biochemical markers assessed in this study, although not significantly altered, did indicate a trend toward improved bone formation. It is worth noting that the participants were early menopausal and did not exhibit osteoporotic conditions, possibly explaining the absence of statistically significant bone turnover changes. Future research may benefit from targeting menopausal populations with and without osteopenia to more precisely evaluate the potential of bone enhancing nutraceuticals.

## 5. Conclusions

This study explored the effects of calcium and collagen peptide supplementation on bone mineral density and skin elasticity in menopausal women. The product containing collagen, vitamin D, and calcium derived from Pangasius (giant catfish) was found to significantly improve skin hydration compared to other test groups in menopausal female participants. The combination outperformed other formulas in reducing transepidermal water loss (TEWL), suggesting enhanced skin barrier function. Additionally, all treatment groups receiving collagen, vitamin D, and calcium or their combination exhibited reduced hair loss compared to the control group.

## Figures and Tables

**Table 1:** The general characteristics and results of body composition analysis of the study participants. The parameters include standard weight, lean body mass (L.B.M.), muscle mass or soft lean mass (SLM), total body water (TBW), protein, and mineral content.

Groups	Month	Height	Weight	Age	Standard of Weight	Lean Body Mass (L.B.M.)	Soft Lean Mass (S.L.M.)	Total Body Water (T.B.W.)	Protein	Minerals
G01	0	155 ± 5.83	56.4 ± 10.15	52 ± 3.89	52.6 ± 3.95	38.4 ± 5.75	35.8 ± 5.36	28.2 ± 4.20	7.52 ± 1.17	2.6 ± 0.41
	2	154 ± 5.97	55.5 ± 10.13	53 ± 3.84	52.5 ± 4.05	37.4 ± 5.48	34.9 ± 5.10	27.6 ± 3.99	7.32 ± 1.12	2.6 ± 0.40
	6	154 ± 5.82	56.5 ± 10.72	53 ± 3.91	52.5 ± 3.94	37.7 ± 5.22	35.2 ± 4.90	27.8 ± 3.85	7.4 ± 1.05	2.6 ± 0.35
G02	0	153 ± 6.20	56 ± 10.53	54 ± 3.31	51.7 ± 4.15	37.4 ± 5.21	34.8 ± 5.21	27.5 ± 3.84	7.3 ± 1.05	2.5 ± 0.34
	2	153 ± 6.33	56.6 ± 9.90	55 ± 2.79	51.8 ± 4.23	36.5 ± 5.20	34.2 ± 4.61	27.1 ± 3.60	7.1 ± 1.05	2.5 ± 0.35
	6	153.1 ± 6.35	55.6 ± 9.82	54.6 ± 3.44	51.6 ± 4.26	37.0 ± 6.13	34.6 ± 5.59	27.3 ± 4.35	7.2 ± 1.24	2.5 ± 0.62
G03	0	153 ± 5.42	57.8 ± 8.52	54 ± 3.92	51.3 ± 3.65	37.5 ± 4.49	34.9 ± 4.20	27.6 ± 4.20	7.3 ± 0.91	2.6 ± 0.31
	2	154 ± 4.88	56.2 ± 8.41	54 ± 4.01	52.0 ± 3.33	37.6 ± 4.42	35.0 ± 4.14	27.7 ± 3.26	7.4 ± 0.88	2.6 ± 0.29
	6	152.9 ± 5.30	58.3 ± 8.03	54.1 ± 4.17	51.5 ± 3.57	37.4 ± 4.49	34.9 ± 4.21	27.6 ± 3.32	7.3 ± 0.92	2.6 ± 0.28
G04	0	153 ± 4.96	53.0 ± 7.54	55 ± 3.41	51.3 ± 3.30	35.5 ± 3.70	33.0 ± 3.50	26.1 ± 2.75	6.9 ± 0.76	2.5 ± 0.22
	2	153 ± 5.20	52.6 ± 7.74	56 ± 3.45	51.2 ± 3.46	35.1 ± 4.23	33.1 ± 4.16	26.1 ± 3.32	6.9 ± 0.85	2.3 ± 0.61
	6	152.8 ± 4.98	53.7 ± 8.07	56.1 ± 3.18	51.4 ± 3.32	35.9 ± 4.14	33.4 ± 3.91	26.4 ± 3.08	7.0 ± 0.83	2.5 ± 0.27

G01 = placebo group; G02 = calcium + vitamin D group; G03 = collagen group; G04 = calcium + vitamin D + collagen group.

**Table 2:** The body composition analysis focusing on muscle and fat analysis, obesity, analysis, and abdominal obesity analysis of the study participants.

Groups	Month	Muscle/Fat Analysis		Obesity Analysis		Abdominal Obesity Analysis	
		Mass Body Fat (M.B.F.)	Skeletal Muscle Mass (S.M.M.)	Body Mass Index (B.M.I.)	Percentage Body Fat (P.B.F.)	Waist to Hip Ratio (W.H.R.)	Visceral Fat Area (V.F.A.)
G01	0	18.05 ± 5.81	21.45 ± 3.22	23.5 ± 3.15	31.33 ± 6.74	0.84 ± 6.74	75.43 ± 34.17
	2	18.06 ± 5.81	20.92 ± 3.06	23.2 ± 3.14	31.85 ± 6.67	0.84 ± 0.06	78.52 ± 35.28
	6	18.8 ± 6.96	21.1 ± 2.93	23.6 ± 3.51	32.1 ± 9.13	0.86 ± 0.07	87 ± 38.60
G02	0	18.7 ± 6.17	20.9 ± 2.93	23.8 ± 3.99	32.6 ± 6.52	0.9 ± 0.07	85.3 ± 52.13
	2	20.0 ± 6.49	20.5 ± 2.77	24.0 ± 3.80	34.8 ± 6.56	0.9 ± 0.08	104.6 ± 58.94
	6	18.5 ± 7.72	20.7 ± 3.35	23.7 ± 3.83	32.6 ± 9.26	0.9 ± 0.08	93.8 ± 57.45
G03	0	20.3 ± 5.22	21.0 ± 2.51	24.7 ± 2.75	34.7 ± 5.00	0.9 ± 0.05	97.5 ± 42.53
	2	18.5 ± 5.60	21.0 ± 2.48	23.7 ± 2.74	32.5 ± 6.27	0.9 ± 0.06	82.3 ± 42.75
	6	20.9 ± 4.93	20.9 ± 2.53	24.9 ± 2.55	35.5 ± 4.81	0.9 ± 0.06	103.6 ± 44.10
G04	0	17.5 ± 4.80	19.6 ± 2.09	22.7 ± 2.46	32.5 ± 5.06	0.9 ± 0.05	80.2 ± 36.53
	2	17.2 ± 4.33	19.8 ± 2.50	22.5 ± 2.50	32.4 ± 4.11	0.9 ± 0.04	77.1 ± 30.85
	6	17.8 ± 5.17	20.0 ± 2.35	22.9 ± 2.62	32.7 ± 5.61	0.9 ± 0.06	83.8 ± 39.95

**Table 3:** The segmental analysis of body composition, including segmental lean mass and segmental fat mass.

Groups	Month	Segmental Lean Mass					Segmental Fat Mass				
		Left Arm S.L.M.	Right Arm S.L.M.	Left Leg S.L.M.	Right Leg S.L.M.	Trunk S.L.M.	Left Arm.B.F.	RightArm M.B.F.	Left Leg M.B.F.	Right Leg M.B.F.	TrunkM.B.F.
G01	0	1.84 ± 0.39	1.90 ± 0.41	5.69 ± 1.00	5.51 ± 1.03	17.94 ± 2.23	1.21 ± 0.11	1.20 ± 0.44	2.67 ± 0.70	2.62 ± 0.72	8.90 ± 3.07
	2	1.80 ± 0.35	1.83 ± 0.37	5.51 ± 1.03	5.35 ± 1.06	17.60 ± 1.96	1.21 ± 0.44	1.21 ± 0.44	2.66 ± 0.71	2.62 ± 0.71	8.92 ± 3.05
	6	1.8 ± 0.38	1.9 ± 0.35	5.5 ± 0.93	5.4 ± 0.98	17.8 ± 1.93	1.3 ± 0.51	1.3 ± 0.52	2.7 ± 0.78	2.7 ± 0.85	9.3 ± 3.68
G02	0	1.78 ± 0.35	1.81 ± 0.36	5.54 ± 1.03	5.36 ± 1.06	17.54 ± 1.90	1.27 ± 0.51	1.27 ± 0.51	2.76 ± 0.81	2.71 ± 0.81	9.17 ± 3.55
	2	1.96 ± 0.94	1.74 ± 0.41	5.78 ± 1.98	5.01 ± 1.33	16.98 ± 2.70	1.69 ± 1.49	1.39 ± 0.52	2.87 ± 0.78	2.76 ± 0.85	10.01 ± 3.50
	6	1.8 ± 0.38	1.8 ± 0.42	5.4 ± 1.16	5.2 ± 1.21	17.6 ± 2.17	1.3 ± 0.59	1.3 ± 0.57	2.7 ± 0.92	2.7 ± 0.95	9.2 ± 4.02
G03	0	1.84 ± 0.33	1.88 ± 0.32	5.36 ± 0.80	5.19 ± 0.84	17.85 ± 1.76	1.39 ± 0.39	1.38 ± 0.39	2.89 ± 0.59	2.86 ± 0.61	10.16 ± 2.85
	2	1.83 ± 0.32	1.88 ± 0.32	5.44 ± 0.80	5.29 ± 0.82	17.81 ± 1.70	1.25 ± 0.42	1.24 ± 0.43	2.69 ± 0.67	2.65 ± 0.69	9.22 ± 2.97
	6	1.8 ± 0.32	1.9 ± 0.31	5.3 ± 0.83	5.1 ± 0.87	18.0 ± 1.69	1.4 ± 0.36	1.4 ± 0.37	2.9 ± 0.56	2.9 ± 0.58	10.5 ± 2.68
G04	0	1.68 ± 0.24	1.72 ± 0.24	5.11 ± 0.75	4.93 ± 0.80	16.95 ± 1.26	1.19 ± 0.36	1.18 ± 0.36	2.56 ± 0.57	2.52 ± 0.59	8.65 ± 2.54
	2	1.93 ± 1.13	1.70 ± 0.28	5.15 ± 1.08	4.73 ± 0.92	16.90 ± 1.44	1.19 ± 0.31	1.17 ± 0.31	2.51 ± 0.52	2.48 ± 0.52	8.86 ± 2.48
	6	1.7 ± 0.31	1.8 ± 0.29	5.1 ± 0.76	4.9 ± 0.79	17.22 ± 1.56	1.2 ± 0.39	1.2 ± 0.39	2.6 ± 0.60	2.5 ± 0.63	8.9 ± 2.76

**Table 4:** The comprehensive evaluation of body composition.

Groups	Month	Comprehensive Evaluation				
		Body Age	Basal Metabolic Rate (B.M.R.)	Calory	Body Cell Mass (B.C.M.)	Total Score
G01	0	53 ± 4.40	1198 ± 124.18	1845 ± 191.21	24.6 ± 3.72	74 ± 7.19
	2	54 ± 4.02	1178 ± 117.47	1814 ± 182.44	24.1 ± 3.62	73 ± 6.33
	6	54 ± 4.06	1185 ± 112.90	1824 ± 173.89	24.3 ± 3.44	74 ± 9.46
G02	0	56 ± 3.39	1176 ± 112.90	1811 ± 173.82	23.9 ± 3.31	73 ± 5.93
	2	57 ± 2.97	1158 ± 112.29	1783 ± 172.84	23.9 ± 3.26	70 ± 6.67
	6	56.3 ± 3.53	1169.7 ± 132.39	1800.8 ± 203.88	23.7 ± 3.58	73.2 ± 11.36
G03	0	56 ± 3.75	1179 ± 96.90	1815 ± 149.28	24.2 ± 2.94	72 ± 5.43
	2	56 ± 2.46	1183 ± 95.49	1821 ± 146.96	24.2 ± 2.88	74 ± 7.48
	6	56.1 ± 3.73	1178.2 ± 97.01	1813.9 ± 149.35	24.2 ± 2.95	70.7 ± 5.52
G04	0	57 ± 3.17	1136 ± 80.02	1749 ± 123.20	22.8 ± 2.38	72 ± 5.35
	2	58 ± 3.17	1133 ± 91.06	1745 ± 140.22	22.9 ± 2.87	72 ± 4.56
	6	57.7 ± 3.23	1144.0 ± 89.29	1761.3 ± 137.57	23.1 ± 2.68	72.3 ± 6.30

**Table 5:** The level of creatinine, ALT, and AST at baseline, 2, and 6 months.

Groups	Month	Creatinine	ALT	AST
		(mg/dL)	(U/L)	(U/L)
G01	0	0.44 ± 0.04	9.8 ± 0.7	13.3 ± 1.0
	2	0.52 ± 0.04	13.9 ± 1.8	17.7 ± 1.5
	6	0.61 ± 0.02	20.1 ± 2.6	24.3 ± 2.0
G02	0	0.52 ± 0.04	14.3 ± 3.5	15.1 ± 2.6
	2	0.60 ± 0.04	20.0 ± 4.6	21.8 ± 3.9
	6	0.66 ± 0.02	23.8 ± 2.5	26.3 ± 2.4
G03	0	0.69 ± 0.03	21.5 ± 1.6	21.1 ± 1.3
	2	0.67 ± 0.03	21.9 ± 2.6	22.7 ± 1.9
	6	0.65 ± 0.02	22.0 ± 2.7	24.2 ± 1.6
G04	0	0.68 ± 0.03	23.4 ± 3.6	26.1 ± 2.4
	2	0.64 ± 0.04	25.8 ± 5.3	26.0 ± 3.0
	6	0.64 ± 0.03	29.8 ± 7.4	28.6 ± 2.8
Normal Range		0.55–1.2	0–34	5.0–34.0

**Table 6:** Level of urine and serum calcium at baseline and 6 months of supplementation and comparison between and within groups.

Calcium Level	Groups				Between Group *p*-Value
	G01	G02	G03	G04	
Random Urine Calcium (mg/dL)					
Baseline	13.43 ± 10.19	8.59 ± 4.59	10.12 ± 4.23	8.77 ± 3.63	0.146
6 months	11.12 ± 6.47	10.22 ± 4.42	9.78 ± 6.98	8.01 ± 3.87	0.473
within group *p*-value	0.914	0.311	0.154	0.794	
Serum Calcium (mg/dL)					
Baseline	9.64 ± 0.32	9.35 ± 0.39	9.55 ± 0.39	9.43 ± 0.40	0.034
6 months	9.31 ± 0.33 *	9.11 ± 0.36	9.13 ± 0.41 *	9.32 ± 0.41	0.206
within group *p*-value	0.004	0.497	0.004	0.766	

* *p* < 0.005.

**Table 7:** Mean value of bone turnover markers at baseline and 6 months of supplementation and comparisons between and within groups.

Bone Formation Markers	Groups				Between Group *p*-Value
	G01	G02	G03	G04	
P1NP (ng/mL)					
Baseline	51.81 ± 18.55	51.01 ± 15.29	46.58 ± 22.88	54.69 ± 21.50	0.529
6 months	52.61 ± 18.47	43.09 ± 17.86	41.85 ± 22.42	50.93 ± 31.97	0.272
Within group *p*-value	0.895	0.137	0.664	0.131	
Osteocalcin (ng/mL)					
Baseline	23.01 ± 8.21	23.09 ± 6.44	23.79 ± 14.11	26.14 ± 15.30	0.910
6 months	23.49 ± 7.73	21.16 ± 6.00	20.77 ± 10.32	24.17 ± 17.18	0.406
Within group *p*-value	0.857	0.342	0.665	0.270	
Bone-specific alkaline phosphatase (BAP) (U/L)					
Baseline	90.26 ± 17.45	106.3 ± 34.20	98.65 ± 29.14	101.8 ± 38.32	0.736
6 months	103.4 ± 23.88	107.6 ± 42.25	100.4 ± 28.55	108.9 ± 45.84	0.919
Within group *p*-value	0.097	0.930	0.993	0.995	

## Data Availability

The data presented in this study are available upon request from the corresponding author due to privacy and ethical restrictions related to patient confidentiality.
